# Low-Dose Aspirin as Primary Prophylaxis for Cardiovascular Events in Rheumatoid Arthritis: An Italian Multicentre Retrospective Study

**DOI:** 10.1155/2019/2748035

**Published:** 2019-05-02

**Authors:** Daniela Iacono, Serena Fasano, Ilenia Pantano, Virginia D'Abrosca, Piero Ruscitti, Domenico Paolo Emanuele Margiotta, Luca Navarini, Nicola Maruotti, Rosa Daniela Grembiale, Francesco Paolo Cantatore, Antonella Afeltra, Roberto Giacomelli, Gabriele Valentini

**Affiliations:** ^1^Rheumatology Section, University of Campania “Luigi Vanvitelli”, Naples, Italy; ^2^Division of Rheumatology, University of L'Aquila, L'Aquila, Italy; ^3^Unit of Rheumatology, Università Campus Bio-Medico di Roma, Rome, Italy; ^4^Rheumatology Clinic, University of Foggia Medical School, Foggia, Italy; ^5^Rheumatology Unit, University of Magna Grecia, Catanzaro, Italy

## Abstract

**Objective:**

To investigate the role of acetylsalicylic acid (ASA) in reducing the incidence of cardiovascular (CV) events in an Italian multicentre rheumatoid arthritis (RA) inception cohort.

**Methods:**

The clinical charts of RA patients consecutively admitted to 4 Italian centres for their 1^st^ visit from November 1, 2000, to December 31, 2015, and followed up till December 2016 were retrospectively investigated for the incidence of CV events. Patients were subdivided into two groups, namely, ASA- and non-ASA-treated groups. The Kaplan–Meier curve and log-rank test were used to investigate differences in event-free survival. Cox regression analysis was carried out to identify factors associated with CV event occurrence.

**Results:**

Seven hundred forty-six consecutive RA patients were enrolled and followed up for a median of 5.6 years (range 2.9–8.9 years). The incidence rate (IR) of CV events was 8/1000 person-years (p-ys) in the overall cohort. The IR of CV events was significantly lower in the ASA-treated group with respect to the non-ASA-treated group (IR 1.7 vs. 11.8/1000 p-ys; *p*=0.0002). The CV event-free rate was longer in ASA-treated patients than in non-ASA-treated patients (log-rank test 12.8; *p*=0.0003). At multivariable analysis, arterial hypertension (HR 9.3) and hypercholesterolemia (HR 2.8) resulted to be positive predictors and ASA (HR 0.09) and hydroxychloroquine (HCQ) (HR 0.22) to be negative predictors.

**Conclusion:**

The IR of CV events in our Italian multicentre cohort was lower than that reported in other European and non-European cohorts. Low-dose ASA may have a role in the primary prophylaxis of CV events in RA patients.

## 1. Introduction

Rheumatoid arthritis (RA) is a chronic systemic inflammatory disorder associated with increased mortality from all-causes and in particular from cardiovascular disease (CVD). Actually, myocardial infarction (MI) and stroke are recognized as leading causes of mortality in patients with RA [1]. Nevertheless, the pathophysiological mechanism underlying the increased CV risk in RA patients is not fully understood. In point of fact, traditional CV risk factors do not fully explain the increased incidence of CV events, observed in these patients [2]. Therefore, RA-associated CV risk seems to be the consequence of the combined effects of chronic systemic inflammation (included platelet activation) and increased traditional CV risk factors and of the treatment with disease-modifying antirheumatic drugs (DMARDs), corticosteroids, and nonsteroidal anti-inflammatory drugs [3–5].

We have recently demonstrated that low-dose acetylsalicylic acid (ASA) and hydroxychloroquine (HCQ) decreased the incidence of CV events in patients with systemic lupus erythematosus (SLE), who are at high risk for atherosclerosis [6, 7].

On this basis, we undertook the present retrospective study to investigate, the role, if any, of ASA in reducing CV morbidity. To that aim, we investigated the Italian multicentre RA cohort, from 4 GIRRCS (*Gruppo Italiano di Ricerca in Reumatologia Clinica e Sperimentale*) centres.

## 2. Methods

### 2.1. Patients

From our database, which includes RA patients consecutively admitted for their 1^st^ visit, from November 1, 2000, to December 31, 2015, to 4 GIRRCS tertiary centres (Academic Rheumatology Units of Naples, l'Aquila, Rome, and Foggia), we selected patients with the following criteria:

- Those who at the first visit satisfied 2010 American College of Rheumatology/European League Against Rheumatism (ACR/EULAR) criteria for RA [8]
- Those who at the first visit had not experienced any prior CV event
- Those who were subsequently assessed at least annually during follow-up

The duration of follow-up was defined as the time from the first visit (baseline visit) to the first CV event or to the last observation in patients without any thrombotic event.

A written informed consent had been obtained by each patient at admission and during follow-up for any new treatment, according to the Declaration of Helsinki. The study was approved by the Ethics Committee of the University of Campania “Luigi Vanvitelli” (CE 278).

### 2.2. Clinical and Laboratory Data

Our database contains information about each patient from admission to throughout follow-up and includes sex, age, disease duration (in years, from onset), autoantibodies profile (serum rheumatoid factor, RF test, cutoff 20 units/mL and anti-citrullinated cyclic peptide antibodies, ACPA, ELISA test, cut off 25 units/mL), disease activity (assessed by Simplefied Disease Activity Index (SDAI)) [9], disability (assessed by the Health Assessment Questionnaire-Disability Index (HAQ-DI)) [10], extra-articular manifestations and radiological features (erosions and joint narrowing), and smoking status (previous/current use of at least one cigarette/day). Concomitant comorbidities and concomitant treatments, as derived from medical records, had been noticed at baseline and from 6-monthly to yearly thereafter. Each patient was investigated for arterial hypertension (prior/ongoing and/or antihypertensive therapy use), diabetes mellitus (fasting glucose level >126 mg/dL in at least two tests and/or ongoing treatment with insulin or oral hypoglycaemic agents), hypercholesterolemia (total cholesterol, TC > 200 mg/dl and/or low-density lipoprotein, LDL > 130 mg/dl and/or high-density lipoprotein, HDL < 40 mg/dl and/or ongoing treatment for hypercholesterolemia), and obesity (body mass index (BMI) > 30 kg/m^2^) [11].

Follow-up intervening treatments, i.e., biological and nonbiological DMARDs (methotrexate, leflunomide, sulfasalazine, and hydroxycloroquine), steroids, and statin use, were registered. Moreover, ASA treatment was recorded if prescribed at any time. In this regard, ASA therapy is currently administered to patients taking glucocorticoids, admitted to the Naples Unit, while in all other centres, ASA was prescribed only to patients with a high CV risk as assessed by traditional risk factors (in both cases, it was not administered to patients in whom it was contraindicated or it was stopped to those experienced side effects) [12]. Patients were then subdivided into two groups, namely, ASA- and non-ASA-treated, considering any patients undergoing ≥1 year ASA treatment as an ASA treated subject.

### 2.3. Outcome Variables

At each visit, any new-onset CV event was recorded. A CV event was defined as the presence of at least one of the following [13]:

1. Ischemic heart disease (IHD), including angina pectoris (confirmed by exercise stress test) or MI (confirmed by electrocardiography and cardiac enzymes)
2. Ischemic cerebrovascular disease (ICVD), including transient ischemic attack (TIA) or stroke supported by an imaging procedure (i.e., computed tomography angiography or magnetic resonance angiography)
3. Ischemic peripheral vascular disease (IPVD): intermitted claudication or peripheral arterial thrombosis, confirmed by an imaging procedure (angiography or Doppler flow studies)

Any intervening event, as defined above, was recorded, and the diagnosis was confirmed by hospital discharge records and/or specific laboratory and diagnostic examinations (i.e., cerebral or myocardial imaging techniques, such as central nervous system computed tomography or magnetic resonance, echocardiography, or myocardial scintigraphy). Causes of death were identified from clinical records, hospital discharge or, when unavailable, by contacting the patient's relatives and obtaining from them written information (i.e., patient's general practitioner report).

On 31 December 2016, the incidence of CV events during follow-up and the vital status of each patient was recorded. Demographic, clinical features and incidence rate of CV events were compared between the two groups.

### 2.4. Statistical Analysis

Continuous variables were analyzed with Student's unpaired *t*-test or with the Mann–Whitney *U* test as appropriate. The chi-squared test or Fisher's exact test was applied for categorical variables. The incidence of CV events was calculated as incidence rate (IR: number of events/person years of observation). Kaplan–Meier curves and the log-rank test were used to analyze differences in event-free survival. Univariable Cox regression analysis served to identify factors associated with CV event occurrence in the overall cohort. The factors found to be significant in univariable analysis were entered in the multivariable stepwise model.

We also derived a propensity score to account for the lack of randomization of ASA treatment. Using logistic regression, we found the predicted probability for the two different groups (ASA vs. non-ASA) using the following confounders: age > 60, smoke, hypertension, hypercholesterolemia, cumulative dose of steroids, and treatment centre. These propensity scores were then used as covariates in a Cox proportional hazards model to establish the relationship between ASA use and CV events.

A value of *p* < 0.05 was considered significant. Analysis was performed with MedCalc, version 12.7.0.0.

## 3. Results

### 3.1. Baseline Data

Seven-hundred forty-six consecutive patients were admitted during the study period.

Table 1 shows epidemiological, serological, and clinical features of the 746 patients of our cohort. Most patients were women (84.8%), with a mean (±standard deviation, SD) age of 59.5 (±13) years and a median disease duration of 11.9 years (interquartile range, IQR 7.37–18). As far as disease features are concerned, 436 patients (58.9%) were positive for RF; 371 patients (52%) were positive for ACPA; 429 patients (60%) had an erosive disease, while 86 patients (11.9%) presented extra-articular manifestations.

As assessed at the first visit, the median SDAI was 14 (IQR 7.2–22.9) and the median HAQ-DI was 1 (IQR 0.5–1.5).

Three-hundred twenty-five patients (45%) were smokers, 367 patients (49.5%) were affected by arterial hypertension, 96 (13.5%) suffered from diabetes, 276 (38%) suffered from hypercholesterolemia, and 112 (15%) were obese.

During the follow-up, all the patients had been managed according to the Treat to Target Strategy; in particular, 456 patients had been treated with biological DMARDs, as we expected in a tertiary centre, with or without conventional synthetic DMARDs (cs DMARDs) and 87% of whole cohort had been treated with steroids (mean cumulative dose: 1.08 g). Furthermore, 149 patients (19.2%) were treated with statins.

### 3.2. Follow-Up Data and CV Events

Patients were followed up for a median of 5.6 years (IQR 2.9–8.9). On 31 December 2016, 33 patients were lost to follow-up (4.4%). These patients were contacted by phone to ascertain vital status and the potential occurrence of CV events. Out of them, 4 patients were died, one for CV events (IMA) and 3 for other causes (2 for respiratory disease and 1 for cancer). At that time, we recorded 38 CV events: 29 MI, 4 stroke, 1 unstable angina, 1 heart failure, 2 atherosclerotic peripheral ischemia, and 1 death due to CV cause.

The IR of CV events in the overall cohort was 8/1000 person-years (38 events/4720 person-years).

### 3.3. ASA Role

Patients were then subdivided into two groups, namely, ASA-treated (242 patients) and non-ASA-treated (504 patients). Patients in the ASA group showed an older age, longer disease duration, higher prevalence of RF, ACPA, erosions, and higher HAQ. Regarding traditional risk factors, patients treated with ASA were more likely to suffer from arterial hypertension, diabetes, hypercholesterolemia, and obesity. On the other hand, SDAI were lower in the ASA group. As far as treatments during follow-up, ASA-treated patients showed a higher prevalence of treatments with methotrexate (MTX) and HCQ and as expected with steroids, as compared to the non-ASA group. As far as cardiovascular events are concerned, only three events occurred in the ASA group (3 events/1758 person-years) vs. 35 in the non-ASA group (35 events/2961 person-years). The IR of CV events was significantly lower in the ASA-treated group with respect to the non-ASA-treated group (IR ASA (1.70/1000) vs. IR non-ASA group (11.8/1000) person-years; *p* = 0.0002).

**Figure 1:**
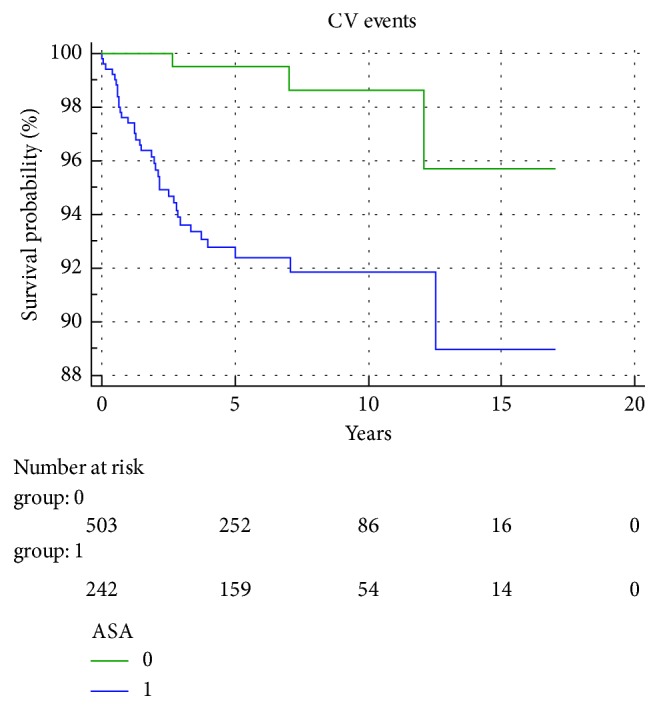
Kaplan–Meier curve: CV event-free rate for ASA-treated group vs. non-ASA-treated group. CV: cardiovascular; ASA: acetylsalicylic acid.

Furthermore, at the Kaplan–Meier curve, the CV event-free rate was higher in ASA-treated patients than in non-ASA-treated patients (log-rank test 12.8; *p* = 0.0003) (Figure 1).

Out of the 242 patients taking ASA, four patients (1.6%) developed menorrhagia, six (2.5%) epigastric pain, and one (0.4%) mild thrombocytopenia, but none of them discontinued ASA.

### 3.4. Predictors of CV Events

Age at first visit, SDAI > 11, arterial hypertension, hypercholesterolemia, and statins resulted to be independent predictors of CV events in univariable analysis as investigated by Cox regression analysis. As far as statins, we think that this result depends on a confounding for indication bias as statins have been prescribed to patients with hypercholesterolemia. On the other hand, biological treatment, HCQ, and ASA treatment were found to have a protective role (Table 2). At multivariable stepwise analysis the independent predictors of CV events were age at first visit (HR 2.82, 95% CI: 1.06–7.49; *p* = 0.004), arterial hypertension, and hypercholesterolemia (HR 9.11, 95% CI: 2.08–39.84; *p* = 0.003 and HR 3.15, 95% CI: 1.25–7.88; *p* = 0.014) as positive predictors; ASA treatment and HCQ treatment as negative predictors (HR 0.09, 95% CI: 0.02–0.37, *p* = 0.0009 and HR 0.21, 95% CI: 0.06–0.71, *p* = 0.012).

After adjustment for propensity score, results were very similar for ASA treatment: HR 0.09, 95% CI 0.02–0.39, *p* = 0.001.

Furthermore, we included in regression models the four treatment centres to account for variations in patients and treatment approaches by the study site. We could not find any significant differences at multivariable analysis (HR 1.29, 95% CI: 0.88–1.89, *p* = 0.188).

## 4. Discussion

We carried out a retrospective analysis of the rate of CV events in 746 patients consecutively admitted to 4 GIRRCS centres, who, at admission, had not experienced any CV event. The IR of CV events in our cohort was significantly lower in the ASA-treated with respect to the non-ASA-treated group (IR ASA group (1.70/1000) vs. IR non-ASA group (11.8/1000) person-years; *p* = 0.0002), and CV event-free rate was higher in ASA-treated than in non-ASA-treated patients (log-rank test 12.8; *p* = 0.0003). These results might depend on the lower disease activity and the higher prevalence of MTX and HCQ-treated patients in the ASA-treated with respect to the non-ASA-treated group. Nevertheless, the higher prevalence of steroid-treated patients, the older age and the longer disease duration, the higher prevalence of RF and ACPA positivity, erosions, arterial hypertension, diabetes, hypercholesterolemia, and obesity in the ASA group seem to indicate a protective role of ASA itself. Actually, ASA intake resulted to be an independent protective factor at multivariable analysis (HR 0.09; 95% CI: 0.02–0.37; *p* = 0.0009), whereas the presence of arterial hypertension and hypercholesterolemia (HR 9.11, 95% CI: 2.08–39.84; *p* = 0.003 and HR 3.15, 95% CI: 1.25–7.88; *p* = 0.014) was independent predictive factors of CV events. These latter results confirm the already reported role of arterial hypertension and hypercholesterolemia as risk factors for CV disease in RA patients [14]. Intriguingly, smoke was not found to exert a promoting role of CV events in our RA cohort. We are inclined to think it depends on a reporting bias; the smoking habit has been reported in only 397 patients from the overall cohort. Finally, a high percentage of our patients were treated with bioDMARDs, this feature depending on the tertiary role of the 4 centres. In any case, the absence of significant difference in the use of bioDMARDs between ASA and non-ASA-treated patients makes a role of these drugs on our results unlikely.

As far as general population, the role of ASA in decreasing the incidence of CV events is debated [15]. Clinical benefits of aspirin for secondary prevention of CV events are well established. However, its use in primary CV prevention remains controversial [16].

The most recent meta-analysis about primary CV prevention pointed out a modest beneficial effect, particularly in older adults as confined to MI [17]. In Italy, it is recommended in patients at high CV risk, like those with RA who are not at increased risk of bleeding [18]. On the other hand, a recent meta-analysis on the role of ASA in the primary prevention of CV events in patients with diabetes did not point out a definite role in the prophylaxis of a first atherosclerotic event or mortality [19]. In conclusion, no agreement has been reached.

As far as RA, in 1978, Linos et al. reviewed clinical charts of high-dose ASA-using RA patients and compared the incidence of CV events with that detected in the general population from Rochester County [20]. These authors failed to find any difference between the 2 groups and interpreted the result as a proof of the absence of any CV protective ASA effect. However, in 1978, despite the previous report by Cobb et al. pointing out an increased mortality by CV events in RA patients [21], accelerated atherosclerosis was not yet recognized as a distinct aspect of the disease [22].

As this aspect is well documented at present, detecting in RA patients an incidence of CV events similar to that in the general population could be regarded as a support to the role of ASA in the prophylaxis of CV events in RA. On the other hand, low-dose ASA in RA patients, using chronic nonsteroidal anti-inflammatory drugs (NSAIDs) and esomeprazole, was not reported to affect the risk of major NSAID toxicity and major adverse CV events [23]. However, our study was not designed for this purpose, and in our cohort, patients were taking neither NSAIDs nor cyclooxygenase-2 inhibitors. Moreover, Durán et al. found no protective role of ASA as primary prophylaxis in a small group of RA patients. Nevertheless, in this study, they included subjects ≥60 years old (mean age 73.5), i.e., significantly older than those from our cohort (mean age 59.5) [24].

We also detected a significant protective role of HCQ for CV events occurrence in RA (HR 0.23, 95% CI: 0.067–0.77; *p* = 0.0172). In that regard, recent evidences in the literature demonstrated that HCQ has a positive impact on metabolic and cardiovascular outcomes in patients with RA, both by decreasing modifiable factors for CVD, namely, lipid profile, diabetes incidence, and glycosylated hemoglobin level and by decreasing the incidence of CV events [25]. Moreover, Sharma et al. have recently studied the association of HCQ use with incident cardiovascular disease (CVD) in a retrospective inception cohort of RA patients, reporting a 72% reduction in the risk of CVD in HCQ users [26]. We cannot rule out a concomitant role of HCQ in reducing the cardiovascular risk of ASA-treated patients. Nevertheless, the significance of Cox regression analysis points out an independent role of ASA.

Our study has some limitations. First of all, it is an observational retrospective study even if patients were prospectively enrolled. Secondly, the IR of CV events recorded in our cohort was 8/1000 person-years (38 events/4720 person-years), that is lower than that reported in other European and non-European cohorts [3]. The small number of CV events in the ASA group underlines the need to investigate the role of low-dose ASA in the prophylaxis of CV events in RA patients from countries with a higher burden of CV disease.

## 5. Conclusion

Our study suggests that low-dose ASA may have a role in the primary prophylaxis of CV events in RA patients. Further larger prospective studies are needed.

## Figures and Tables

**Table 1:** Baseline features and treatment during follow-up of the overall cohort.

Baseline features	
Sex: F/M ratio (%)	633/113 (84.8%)
Age (years)	
Median (IQR)	60.9 (52–68.6)
Mean ± SD	59.5 ± 13
Disease duration, years from onset	
Median (IQR)	11.9 (7.37–18)
RF+/*−*, *n* (%)	436/304 (58.9%)
ACPA+/*−*, *n* (%)	371/349 (52%)
Erosion+/*−*, *n* (%)	429/282 (60%)
SDAI baseline	
Median (IQR)	14 (7.2–22.9)
HAQ-DI baseline	
Median (IQR)	1 (0.5–1.5)
Smoke+/*−*, *n* (%)	325/397 (45%)
Hypertension+/*−*, *n* (%)	367/379 (49%)
Diabetes+/*−*, *n* (%)	96/650 (13%)
Hypercholesterolemia+/*−*, *n* (%)	276/449 (38%)
Obesity+/*−*, *n* (%)	112/631 (15%)

Treatment during follow-up	

Anti-TNF, *n* (%)	393/353 (52.6%)
Mean duration (years)	5.23
Non-anti-TNF-bDMARDs, *n* (%)	209/536 (28%)
Mean duration (years)	3.68
MTX+/*−*, *n* (%)	649/97 (87%)
Mean duration (years)	6.16
Other csDMARDs+/*−*, *n* (%) Mean duration (years)	Leflunomide 182/564 (24.4%); yrs 4.06 Sulfasalazine 127/617 (17%); yrs 2.8 Hydroxychloroquine 288/458 (38.6%); yrs 4.25
Low-dose steroids (2.5–5 mg), *n* (%)	651/95 (87.2%)
Mean duration (years)+	6.88
Cumulative dose of steroids (g)	
Mean ± SD	1.08 ± 1.07
Statin+/*−*, *n* (%)	149/494 (19.2%)
Mean duration (years)	5.15

IQR: interquartile range; SD: standard deviation; RF: rheumatoid factor; ACPA: anti-citrullinated cyclic peptide antibodies; SDAI: Simplified Disease Activity Index; HAQ-DI: Health Assessment Questionnaire-Disability Index; TNF: tumor necrosis factor; csDMARDs: conventional synthetic disease-modifying antirheumatic drugs; bDMARDs: biological disease-modifying antirheumatic drugs; MTX: methotrexate; ASA: acetylsalicylic acid.

**Table 2:** Univariate analysis: factors associated with cardiovascular event occurrence.

Features	HR (CI)	*p*
Sex (F)	1.21 (0.43–3.09)	0.693
Age > 60 years	4.75 (1.98–11.35)	**0.0005** ^*∗*^
RF+	0.91 (0.52–1.62)	0.752
ACPA+	1.80 (0.92–3.52)	0.08
Erosion	1.11 (0.56–2.20)	0.76
SDAI > 11	2.40 (1.03–5.59)	**0.0004** ^*∗*^
Smoke	0.58 (0.28–1.18)	0.1334
Hypertension	19.3 (4.65–80.18)	**0.0001** ^*∗*^
Diabetes	2.14 (1.01–4.52)	0.046
Hypercholesterolemia	5.13 (2.33–11.3)	**<0.0001** ^*∗*^
Obesity	1.29 (0.56–2.95)	0.543
Biological treatment	0.42 (0.22–0.82)	**0.01** ^*∗*^
HCQ	0.12 (0.04–0.39)	**0.0005** ^*∗*^
MTX	0.99 (0.98–1.01)	0.940
Statins	3.68 (1.88–7.23)	**0.0001** ^*∗*^
ASA	0.15 (0.05–0.51)	**0.002** ^*∗*^
Cumulative dose of steroids	1 (0.99–1)	0.186

HR: hazard ratio; CI: confidence interval; RF: rheumatoid factor; ACPA: anti-citrullinated cyclic peptide antibodies; SDAI: Simplified Disease Activity Index; HAQ-DI: Health Assessment Questionnaire-Disability Index; HCQ: hydroxychloroquine; MTX: methotrexate; ASA: acetylsalicylic acid. ^*∗*^A *p* value < 0.05 was considered for factors associated with cardiovascular event occurrence (positive predictor if HR > 1, negative if HR < 1).

## Data Availability

The data that support the findings of this study are available on request from the corresponding author. The data are not publicly available because of the information that could compromise the research participant privacy/consent.

## Abbreviations

RA: Rheumatoid arthritis
CVD: Cardiovascular disease
MI: Myocardial infarction
DMARDs: Disease-modifying antirheumatic drugs
ASA: Acetylsalicylic acid
PGI/Tx2: Prostaglandin I/thromboxane A2
SLE: Systemic lupus erythematosus
GIRRCS: Gruppo Italiano di Ricerca in Reumatologia Clinica e Sperimentale
ACR/EULAR: American College of Rheumatology/European League Against Rheumatism
TIA: Transient ischemic attack
RF: Rheumatoid factor
ACPA: Anti-citrullinated cyclic peptide antibodies
SDAI: Simplified Disease Activity Index
HAQ-DI: Health Assessment Questionnaire-Disability Index
TC: Total cholesterol
LDL: Low-density lipoprotein
HDL: High-density lipoprotein
BMI: Body mass index
IR: Incidence rate
SD: Standard deviation
IQR: Interquartile range
MTX: Methotrexate
HCQ: Hydroxychloroquine
HR: Hazard ratio
CI: Confidence interval
NSAID: Nonsteroidal anti-inflammatory drug.

## Additional Points

*Significance and Innovations.* (i) CV morbidity and mortality are significantly greater in RA. (ii) Cardiovascular morbidity is lower in ASA-treated RA patients. (iii) ASA may have a role as primary prophylaxis of CV events in RA.
